# Sleep recalibrates homeostatic and associative synaptic plasticity in the human cortex

**DOI:** 10.1038/ncomms12455

**Published:** 2016-08-23

**Authors:** Marion Kuhn, Elias Wolf, Jonathan G. Maier, Florian Mainberger, Bernd Feige, Hanna Schmid, Jan Bürklin, Sarah Maywald, Volker Mall, Nikolai H. Jung, Janine Reis, Kai Spiegelhalder, Stefan Klöppel, Annette Sterr, Anne Eckert, Dieter Riemann, Claus Normann, Christoph Nissen

**Affiliations:** 1 Department of Psychiatry and Psychotherapy, University Medical Center Freiburg, Hauptstrasse 5, 79104 Freiburg, Germany; 2 Department of Pediatrics, Technische Universität München, Arcisstrasse 21, 80333 Munich, Germany; 3 Department of Neurology, University Medical Center Freiburg, Breisacher Strasse 64, 79106 Freiburg, Germany; 4 Department of Psychology, University of Surrey, Guildford, Surrey GU2 7XH, UK; 5 Neurobiology Lab for Brain Aging and Mental Health, Transfaculty Research Platform University of Basel, Psychiatric University Clinics Basel, Wilhelm Klein-Strasse 27, 4012 Basel, Switzerland

## Abstract

Sleep is ubiquitous in animals and humans, but its function remains to be further determined. The synaptic homeostasis hypothesis of sleep–wake regulation proposes a homeostatic increase in net synaptic strength and cortical excitability along with decreased inducibility of associative synaptic long-term potentiation (LTP) due to saturation after sleep deprivation. Here we use electrophysiological, behavioural and molecular indices to non-invasively study net synaptic strength and LTP-like plasticity in humans after sleep and sleep deprivation. We demonstrate indices of increased net synaptic strength (TMS intensity to elicit a predefined amplitude of motor-evoked potential and EEG theta activity) and decreased LTP-like plasticity (paired associative stimulation induced change in motor-evoked potential and memory formation) after sleep deprivation. Changes in plasma BDNF are identified as a potential mechanism. Our study indicates that sleep recalibrates homeostatic and associative synaptic plasticity, believed to be the neural basis for adaptive behaviour, in humans.

## Abstract (web summary)

Sleep deprivation is believed to lead to homeostatic increases in synaptic strength and reduced inducibility of associative LTP, based mainly on findings from animal studies. Here, Kuhn *et al*. demonstrate similar sleep-dependent synaptic plasticity changes in humans along with altered plasma BDNF levels.

## Graphical abstract

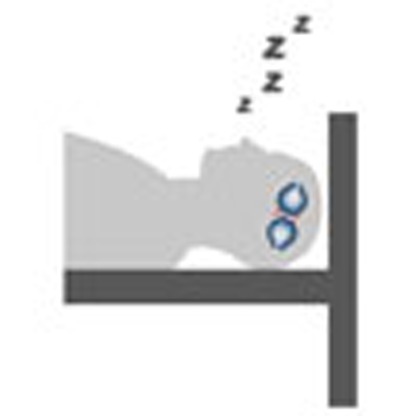

The activity-dependent refinement of transmission across single synapses (associative plasticity) and the up- and downscaling of overall synaptic strength (homeostatic plasticity) represent basic mechanisms for neural network function and adaptive behaviour^1^. Sleep has been shown to strongly modulate synaptic plasticity^2^, but its effects on the interplay of homeostatic and associative plasticity remain to be further characterized.

The synaptic homeostasis hypothesis of sleep–wake regulation posits that downscaling (that is, the decrease in strength) of synapses that have been potentiated towards saturation during wakefulness denotes a vital function of sleep^3^,^4^. Sleep-dependent synaptic desaturation is thought to result in an improved signal-to-noise ratio and a renewed capacity for the encoding of new information through associative plasticity. This hypothesis is supported by molecular and electrophysiological evidence from animal studies^5^. Particularly, major markers of synaptic strength show a wake-dependent rise and a sleep-dependent decline, such as the slope of the local field potential elicited by electrical stimulation in the rat cortex^6^, the level of GluR1-containing AMPA receptors in the rat hippocampus and cortex^6^, cortical spine density in adolescent mice^7^, and the number or size of synapses in different neuronal circuits in flies^8^,^9^. In humans, molecular mechanisms of synaptic homeostasis are not directly accessible, but non-invasive (indirect) markers indicate net synaptic potentiation during wakefulness and desaturation during sleep. For instance, the slope of the electroencephalographic (EEG) response to transcranial magnetic stimulation (TMS) that shares properties with local field potentials in animal studies increases with time awake and resets after sleep^10^. Further, EEG theta activity, a marker for homeostatic sleep pressure (net synaptic potentiation) in rats^11^ and humans^12^, increases with time awake. Also other neurophysiological markers point to an increase in cortical excitability (that is, the propensity for firing) and overall synaptic strength, such as an increase in short-interval intracortical facilitation and a decrease in short-interval intracortical inhibition in M1 after sleep deprivation^10^,^13^,^14^,^15^.

These sleep–wake dependent homeostatic modifications of net synaptic strength appear to refine the set-point for the induction of input-specific associative plasticity at single synapses. Particularly, Hebbian long-term potentiation (LTP) of glutamatergic synaptic transmission is a key mechanism of associative plasticity and a molecular correlate for learning and memory^16^. LTP is a positive feedback process with a tendency to saturation^17^, which appears to be prevented by sleep-dependent homeostatic desaturation. Consistently, the induction of LTP via high-frequency electrical stimulation in the motor cortex of rats is partially occluded after prolonged wakefulness and restored after sleep^6^. In humans, hippocampal activity assessed by functional magnetic resonance imaging is reduced during the encoding of episodic memory after sleep deprivation along with a deficit in the formation of new memories as a behavioural correlate of activity-dependent synaptic plasticity^18^. Yet memory is a complex process including multi-synaptic pathways, and the sleep–wake-dependent interplay of homeostatic and associative synaptic plasticity in humans remains to be further determined. Associative LTP-like plasticity can be non-invasively induced in the human cortex by the brain stimulation protocol paired associative stimulation (PAS)^19^. The PAS-induced LTP-like plasticity shows similar characteristics to LTP in animal slice experiments, namely associativity, input-specificity and dependency on *N*-methyl-D-aspartate receptor functioning^20^.

This study used TMS and EEG to investigate cortical excitability/net synaptic strength (TMS intensity to elicit a predefined amplitude of motor-evoked potential (MEP) and EEG theta activity) and LTP-like plasticity (PAS induced change in MEP and memory formation) in the human cortex. For the first time to our knowledge, our study combines the assessment of indices of homeostatic and associative synaptic plasticity in humans. We demonstrate an increase in cortical excitability/net synaptic strength and a partial occlusion of LTP-like plasticity after sleep deprivation compared with sleep, indicating that sleep recalibrates synaptic plasticity in the human cortex.

## Results

### Increased net synaptic strength after sleep deprivation

TMS and wake EEG were investigated as non-invasive indices of cortical excitability/net synaptic strength in 20 healthy participants in a repeated-measures protocol after one night of sleep and one night of sleep deprivation (counterbalanced order with one-week interval).

**Figure 1:**
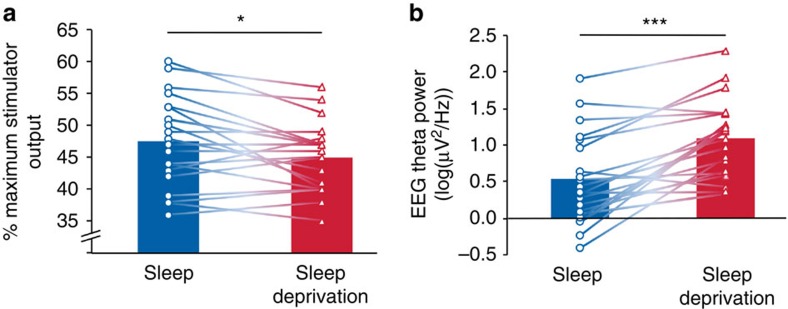
Indices of cortical excitability/net synaptic strength. (**a**) Stimulation intensity of transcranial magnetic stimulation (% maximum stimulator output) was significantly lower after sleep deprivation compared with sleep. (**b**) Theta activity of wake electroencephalography (EEG) was significantly higher after the sleep deprivation compared with the sleep condition. Bars represent means (*n*=20). Individual data lines visualize the change between the two conditions at the single-subject level. Paired-sample *t*-tests were used (two-tailed): *P<0.05, ***P<0.001.

TMS was used to elicit MEPs of the abductor pollicis brevis (APB) muscle of the left hand. The stimulation intensity adjusted to elicit comparable baseline MEP amplitudes before PAS (see Supplementary Table 1) was significantly lower in the sleep deprivation condition compared with the sleep condition (*t*_19_=−2.46, *P*=0.023; Fig. 1a), indicative for an increased cortical excitability after one night of sleep deprivation. There was no difference in the resting motor threshold (RMT) before PAS between the two conditions (sleep deprivation: 35.9±3.7% of maximum stimulator output, MSO; sleep: 36.7±5.5% MSO; *t*_19_=−0.5, *P*=0.624).

**Figure 2:**
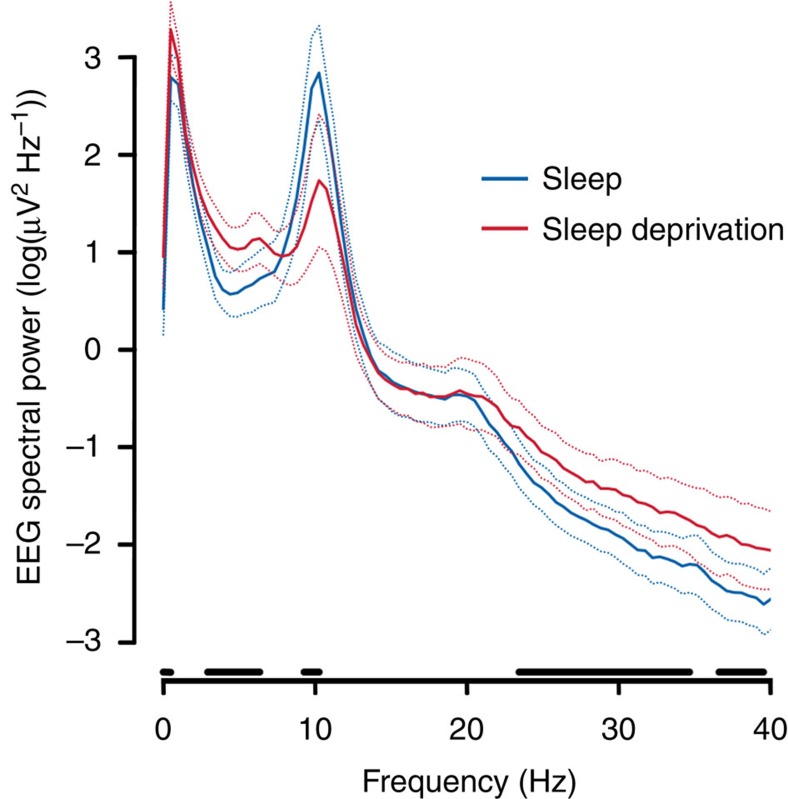
Electroencephalographic (EEG) spectral power. After sleep deprivation, power density in the 3.5–8 Hz (theta) frequency range was significantly higher compared with the sleep condition (*P*<0.05). Exploratory analyses revealed condition effects in other frequency ranges. Solid lines represent means (*n*=20). Dashed lines represent 95% confidence intervals. Paired-sample *t*-tests were used (two-tailed). Horizontal lines indicate significant condition effects (*P*<0.05).

All participants completed a wake EEG protocol to assess power density in the 3.5–8 Hz (theta) frequency range that has been described as a marker for the buildup of the homeostatic process S of sleep regulation in rats^11^ and humans^12^, which in turn has recently been proposed to reflect the level of net synaptic strength during wakefulness^4^. The wake EEG data demonstrated a highly significant increase in EEG theta power after the sleep deprivation compared to the sleep condition (*t*_19_=5.1, *P*<0.001; Fig. 1b). The full EEG spectrum is presented in Fig. 2. As shown, exploratory analyses revealed condition effects in other frequency ranges. However, we restricted the main analysis and interpretation to the EEG theta range.

### Decreased LTP-like plasticity after sleep deprivation

We used PAS and a word-pair task to investigate indices of LTP-like plasticity. The PAS protocol followed standard procedures and consisted of 200 pairs of peripheral and cortical stimuli at a frequency of 0.25 Hz (ref. 19). Peripheral electrical stimulation of the median nerve at the left wrist was followed by TMS of the right primary motor cortex (M1) at the optimal site to elicit MEPs in the left APB muscle with an interstimulus interval (ISI) of 25 ms. At this ISI, the electrical stimulus slightly precedes the TMS pulse in M1 leading to a LTP-like potentiation of synaptic transmission that can be measured as an increase of the TMS-evoked MEP amplitude in the corresponding hand muscle. This PAS-induced LTP-like potentiation shows similar properties to LTP in animal slice experiments^20^.

**Figure 3:**
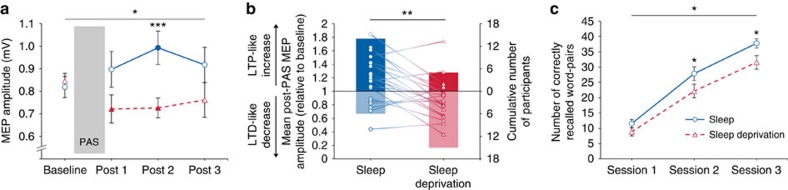
Indices of long-term potentiation (LTP)-like plasticity. (**a**) After sleep deprivation, the inducibility of LTP-like plasticity was significantly impaired compared with the sleep condition, as measured by the increase in motor-evoked potential (MEP) amplitude 2 (post 1), 30 (post 2) and 60 min (post 3) after paired associative stimulation (PAS). Filled symbols indicate a significant (*P*<0.05) increase (after sleep) or decrease (after sleep deprivation) of post-PAS MEP amplitudes referred to baseline. (**b**) Response pattern to the PAS protocol at the single-subject level. Symbols represent the ratio of mean MEP amplitudes post-PAS (averaged across post 1–3) to MEP amplitudes before PAS (baseline) for each subject in both conditions. A ratio >1.0 indicates a LTP-like increase and a ratio <1.0 indicates a long-term depression (LTD)-like decrease in MEP amplitudes after PAS. Bars represent the cumulative number of participants who showed a LTP-like increase or LTD-like decrease in the sleep and the sleep deprivation condition, respectively. (**c**) The number of correctly recalled word-pairs in a declarative memory task was significantly lower after sleep deprivation compared with sleep. Data represent means±s.e.m. (*n*=20). Horizontal lines with asterisks indicate significant rm-ANOVAs (**a**,**c**) or a significant *χ*^*2*^-test (**b**). Paired-sample *t*-tests were used (two-tailed): *P<0.05, ***P=0.001.

Figure 3a visualizes the observed decrease in LTP-like plasticity induced by PAS after sleep deprivation compared with sleep, as measured by the change in MEP amplitudes. The 2 × 4 repeated measures analysis of variance (rm-ANOVA) showed a significant effect for the factor Condition (*F*_1,19_=7.5, *P*=0.013, *η*_p_^2^*=*0.283), no significant effect for the factor Time (*F*_3,57_=0.37, *P*=0.778, *η*_p_^2^*=*0.019) and a significant Condition × Time interaction (*F*_3,57_=3.5, *P*=0.021, *η*_p_^2^*=*0.156). *Post hoc* paired-sample *t*-tests demonstrated similar baseline MEP amplitudes before PAS and a significantly higher MEP amplitude at post 2 after sleep compared with sleep deprivation (see Supplementary Table 1). This effect was driven by a LTP-like increase in MEP amplitude at post 2 relative to baseline in the sleep condition (*t*_19_=2.6, *P*=0.018), and a decrease in MEP amplitude at post 2 after sleep deprivation (*t*_19_=−2.8, *P*=0.013). Also at post 1, there was a significant decrease in MEP amplitude compared to baseline in the sleep deprivation condition (*t*_19_=−2.2, *P*=0.039). The other contrasts did not reach statistical significance.

To further characterize the response pattern to the PAS protocol at the single-subject level (Fig. 3b), we calculated the ratio of mean MEP amplitudes post-PAS (averaged across post 1–3) to MEP pre-PAS, with a ratio >1.0 indicating a LTP-like increase and a ratio <1.0 indicating a long-term depression (LTD)-like decrease in MEP amplitudes^21^. The distribution of the LTP-like increase and LTD-like decrease after PAS was significantly inverse after sleep and sleep deprivation (Fig. 3b): After sleep, 14 participants (70%) showed an LTP-like increase and six participants (30%) showed an LTD-like decrease, whereas after sleep deprivation only five participants (25%) showed an LTP-like increase and 15 participants (75%) showed an LTD-like decrease (*χ*^2^_1_=8.1, *P*=.004). There was no significant correlation between the baseline MEP amplitude (pre-PAS) and the mean MEP amplitudes post-PAS (sleep: *r*=−0.32, *P*=0.168; sleep deprivation: *r*=−0.25, *P*=0.279).

A word-pair task was used to assess declarative memory as a proxy of synaptic plasticity in a hippocampal-neocortical network^22^. In the word-pair task, 46 semantically unrelated word-pairs were randomly presented on a computer screen. Immediately after the presentation session, participants had to name the second word upon presentation of the first word (cued recall). Three presentation/recall sessions were conducted and the number of correctly recalled words in each session was recorded. Consistent with the notion of a partial occlusion of LTP-like plasticity after sleep deprivation, participants demonstrated reduced memory acquisition in the word-pair task after sleep deprivation. A 2 × 3 rm-ANOVA with the within-subject factors Condition (sleep, sleep deprivation) and Session (sessions 1–3) and the number of correctly recalled word-pairs in each session as the dependent variable revealed significant main effects for the factors Condition (*F*_1,19_=6.8, *P*=0.018, *η*_p_^2^*=*0.262) and Session (*F*_2,38_=377.9, *P*<0.001, *η*_p_^2^*=*0.952), but no significant interaction (*F*_2,38_=2.9, *P*=0.069, *η*_p_^2^*=*0.132). *Post hoc* paired-sample *t*-tests demonstrated nonsignificant performance differences in session 1 (*t*_19_=−1.7, *P*=0.098) and a significantly reduced number of recalled word-pairs in session 2 (*t*_19_=−2.4, *P*=0.026) and session 3 (*t*_19_=−2.8, *P*=0.012) in the sleep deprivation compared with the sleep condition (Fig. 3c).

### Potential modulators of synaptic plasticity

**Figure 4:**
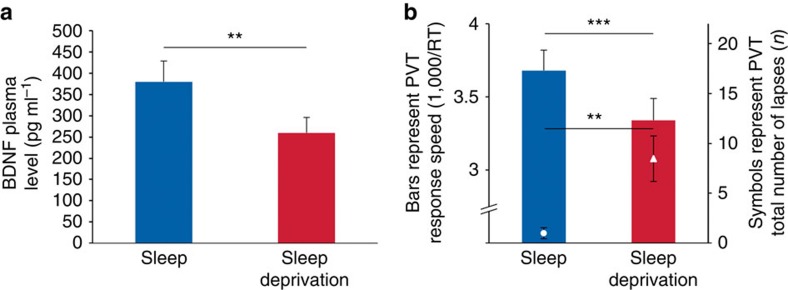
Potential modulators of synaptic plasticity. (**a**) Brain-derived neurotrophic factor (BDNF) plasma level was significantly lower after sleep deprivation compared with sleep. (**b**) After sleep deprivation, the participants were less vigilant, as indicated by a significant reduction in response speed (1,000 ms per reaction time) and a significant increase in the total number of lapses (reaction time ≥500 ms) in the Psychomotor Vigilance Task (PVT). Data represent means±s.e.m. (*n*=20). Paired-sample *t*-tests were used (two-tailed): **P<0.01, ***P<0.001.

To investigate the impact of the sleep and sleep deprivation condition on brain-derived neurotrophic factor (BDNF), cortisol and vigilance, we used paired sample *t*-tests (Fig. 4a,b). The BDNF plasma level was significantly lower after sleep deprivation (259.5±162.8 pg ml^−1^) compared with sleep (379.6±220.1 pg ml^−1^) (*t*_19_=−3.37, *P*=0.003; Fig. 4a).

Salivary cortisol levels at 0600, h (sleep: 18.3±11.6 nmol l^−1^, sleep deprivation: 13.5±8.2 nmol l^−1^) and 0800, h (sleep: 24.5±8.9 nmol l^−1^, sleep deprivation: 24.6±8.8 nmol l^−1^) did not differ between the conditions (for both time points: *P*>0.05; results not visualized). Comparable salivary cortisol levels in the morning after one night of sleep deprivation are consistent with prior studies^23^,^24^.

The Psychomotor Vigilance Task (PVT) was used as a sensitive measure of changes in vigilance based on reaction times^25^. PVT response speed was significantly reduced (*t*_18_=−7.1, *P*<0.001) and the number of lapses was significantly increased (*t*_18_=3.2, *P*=0.005) after the sleep deprivation compared with the sleep condition (Fig. 4b).

To further investigate the relationship between potential modulators of synaptic plasticity (BDNF, cortisol and vigilance) and cortical excitability and LTP-like plasticity, exploratory linear regression was used examining the degree to which the observed changes in TMS intensity (to elicit baseline MEPs of about 1 mV peak-to-peak (SI_1mV_) before PAS), EEG theta activity, MEP amplitude and the number of correctly recalled word-pairs between sleep and sleep deprivation relate to corresponding changes in BDNF plasma level, cortisol level and PVT response speed. Using difference values (sleep–sleep deprivation condition), a linear model was used to predict the difference in each of the four dependent variables using the differences in the three independent variables.

In a first regression model with TMS intensity as the dependent variable, the change in BDNF plasma level was a significant predictor of the change in TMS intensity (*β*=−0.565*, P*=0.011), such that a higher decrease in BDNF plasma level after sleep deprivation was associated with a lower decrease in TMS intensity (lower increase in cortical excitability). The change in cortisol (*β*=−0.256*, P*=0.207) and vigilance (*β*=−0.213*, P*=0.291) did not reach significance. The proportion of the explained variance (*R*^2^) was 44%. In a second, third and fourth regression model with the change in EEG theta activity, the change in MEP amplitudes and the change in correctly recalled word-pairs as the dependent variables, no predictor variable reached significance (for all: *P*≥0.05).

The BDNF genotype analysis revealed 12 val/val homozygotes, seven val/met heterozygotes, and one met/met homozygote participant. To explore whether the BDNF genotype impacts cortical excitability and LTP-like plasticity, we conducted three separate multivariate ANOVAs, one for each condition and one for the change between conditions (using difference values: sleep—sleep deprivation condition), with the between-subject factor Genotype (val/val versus met carriers) and TMS intensity, EEG theta activity and mean MEP amplitude as the dependent variables. There was no significant effect for the factor Genotype (for all: *P*>0.05).

## Discussion

This study demonstrates indices of decreased LTP-like plasticity and increased net synaptic strength in the human cortex after sleep deprivation. As such, our study provides first evidence for a sleep–wake-dependent dissociation of associative and homeostatic synaptic plasticity in humans.

The propensity of the brain to express associative synaptic plasticity, the molecular basis for adaptive behaviour, appears to depend on the prior history of the sleep/wake state. Because synaptic plasticity cannot directly be quantified in humans, we assessed the PAS-induced change in MEP amplitude that shares key properties with LTP *in vitro*, namely associativity, cooperativity and input-specificity^19^,^26^. We demonstrate, for the first time, a partial occlusion of LTP-like plasticity after sleep deprivation in the human cortex. This extends *in vivo*^6^ and *in vitro* studies in rats^27^,^28^,^29^ showing impaired induction of synaptic LTP after sleep deprivation. We further demonstrate a significant deficit in the encoding of declarative memory after sleep deprivation (word-pair task), which is thought to depend on the induction of LTP in a hippocampal-neocortical network^30^. This confirms prior work on declarative memory^18^ and suggests that basic deficits in associative synaptic plasticity after sleep deprivation, as demonstrated by PAS, also translate to the behavioural level.

The synaptic homeostasis hypothesis proposes that homeostatic modifications of net synaptic strength across the sleep–wake cycle define the set-point for the induction of associative plasticity^5^. Our results support this notion by demonstrating markers of increased cortical excitability and net synaptic strength after sleep deprivation. Particularly, we confirm enhanced wake EEG theta activity after sleep deprivation^12^,^31^. This EEG signature has been shown to closely correlate with a major marker of net synaptic strength in humans, that is, EEG slow wave activity during sleep^12^. We further demonstrate increased cortical excitability based on an attenuated stimulation intensity of TMS pulses to elicit a predefined amplitude of MEPs (before PAS) after sleep deprivation. In line with previous work^13^,^14^,^32^,^33^, a marker of axon membrane excitability (RMT) was not affected by sleep deprivation. Future studies are needed to further investigate potential other mechanisms of excitability changes, such as intracortical inhibition. Together, we demonstrate markers of increased excitability along with decreased inducibility of LTP-like plasticity in the human cortex after sleep deprivation.

**Figure 5:**
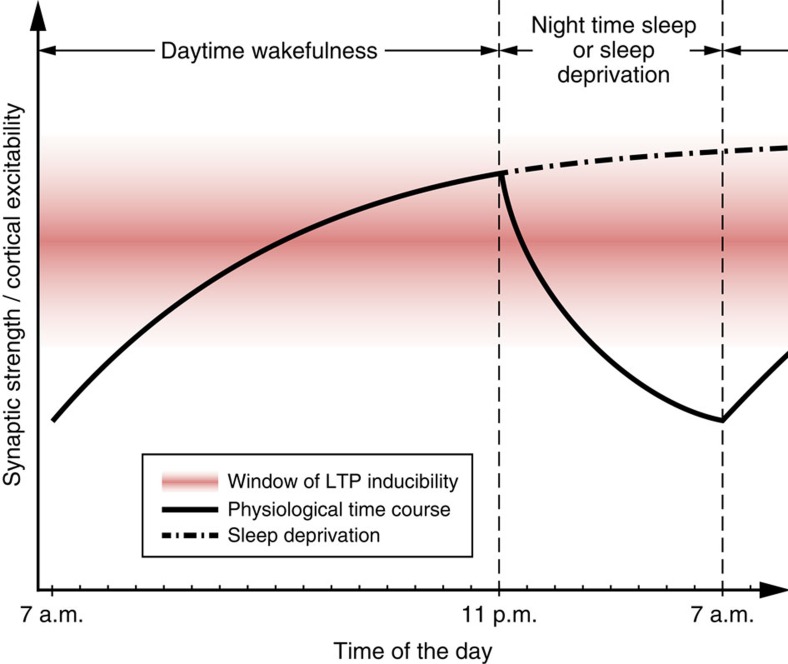
‘Happy medium' model of LTP inducibility across the sleep wake cycle. The figure depicts the proposed interplay between the time course of synaptic strength/cortical excitability (homeostatic plasticity; solid and dotted lines) and the inducibility of associative synaptic long-term potentiation (LTP)-like plasticity (red window). Wakefulness is associated with an upscaling and sleep with a downscaling of net synaptic strength. Sleep deprivation eventually leads to synaptic saturation and deficient LTP inducibility.

Our results, together with prior work, lead us to propose a sleep–wake dependent window of optimal associative plasticity (‘happy medium'), with attenuated inducibility of LTP both directly after sleep (insufficient upscaling) and after extended wakefulness (excessive upscaling and saturation) (Fig. 5). This idea is supported by reduced LTP-like plasticity in the morning and facilitated LTP-like plasticity in the evening (assessed by PAS) in healthy humans^34^. Decreased LTP in rats^6^ and LTP-like plasticity in our study after extended wakefulness is proposed to result from a shift out of the window of optimal associative plasticity.

With regard to potential mechanisms, BDNF plasma levels were significantly decreased after sleep deprivation compared with sleep. Interestingly, we observed that a stronger decrease in BDNF plasma level after sleep deprivation was associated with a smaller increase in cortical excitability as measured by TMS. We speculate that a pronounced decline in BDNF across sustained wakefulness might protect individuals from excessive upscaling and related shortcomings, such as saturation and impaired information processing. Whereas individuals with high BDNF activity outperform those with low activity under standard daytime conditions^35^, this advantage might be inversed after prolonged wakefulness. This could complement our picture of the advantages and disadvantages of high and low BDNF activity. Future studies are needed to further test this speculation. Changes in cortisol and vigilance did not significantly explain the changes in cortical excitability or LTP-inducibility in the current study.

To date, there is an inconclusive debate on how distinct measures of plasticity in different neural networks might relate to each other. Studies indicate that LTP is an omnipresent mechanism of plasticity in the brain^36^. Particularly, LTP-like effects have been observed in several neuronal networks in humans, for example, in the visual cortex, the motor cortex and the hippocampal-neocortical network. However, the paradigms used to induce these effects are highly heterogeneous (presumably with different sensitivities), and the complexity of the networks involved varies from a few synapses to extended neural networks. Thus in many studies, including ours, different measures used to index plasticity did not closely correlate. For example, LTP-like plasticity induced by PAS did not correlate with the performance in a motor learning task^37^,^38^ or verbal learning^39^. Nevertheless, these forms of plasticity probably share LTP at the molecular level as a common mechanism and the development of more sensitive measures is needed.

Of particular note, we observed inverse effects of the PAS protocol after sleep and sleep deprivation (Fig. 3b). MEP amplitudes increased in 70% of the participants after sleep (LTP-like), whereas MEP amplitudes decreased in 75% of the participants after sleep deprivation (LTD-like) using the same PAS protocol. In fact, some evidence indicates that the induction of LTD is facilitated after sleep deprivation in the rat hippocampus^40^,^41^,^42^. One possible explanation is provided by the Bienenstock–Cooper–Munro theory of bidirectional synaptic plasticity. The Bienenstock–Cooper–Munro theory proposes that the threshold for LTP/LTD induction is adjusted to the level of prior postsynaptic activity^43^. Extensive wakefulness might cause high postsynaptic plasticity and shift the likelihood threshold from LTP to LTD. Further studies are needed to test this speculation. Another explanation for facilitated LTD induction after sleep deprivation might be provided by sleep–wake-dependent changes in neural bistability. This refers to switches between on- and off-states of neural populations with an increased incidence of local off-states with increasing sleep pressure (‘local sleep')^44^. After sleep deprivation, presynaptic activity elicited by the TMS stimulus might coincide with a hyperpolarized down-state of the postsynaptic membrane (that could even be triggered by the peripherally induced volley of the PAS protocol), a classic precondition for LTD. However, this latter idea of local sleep cannot explain why PAS effectiveness is higher in the evening than in the morning after normal sleep. Moreover, increased bistability would be expected to result in increased variability between individual MEP responses in the sleep deprivation compared to the sleep condition, which was not the case in the present study. Future studies are needed to disentangle the mechanisms of the observed decrease in MEP amplitudes after sleep deprivation.

The current line of research has important clinical implications. In modern society, chronic sleep loss is a highly prevalent problem. Therefore, it is crucial to further delineate the impact of sleep on healthy performance and underlying mechanisms, such as required for school, work or social life. In addition, further characterization of homeostatic and associative plasticity across the sleep–wake cycle might have interesting implications for neuropsychiatric disorders. For instance, in patients with major depressive disorder, the inducibility of LTP-like plasticity is impaired compared with controls^38^,^45^. Conceivably, therapeutic sleep deprivation, an effective treatment with rapid onset in major depressive disorder, might shift the initially deficient LTP inducibility into a more favourable window of associative plasticity^46^.

In conclusion, this is the first study to demonstrate a sleep–wake-dependent dissociation of homeostatic and associative synaptic plasticity in the human cortex with enhanced cortical excitability and a partial occlusion of associative LTP-like plasticity after sleep deprivation. Further investigation of the interplay of different types of plasticity and its modulation by sleep might represent a significant step towards a better understanding of basic mechanisms of healthy performance and potential alterations in neuropsychiatric disorders.

## Methods

### Participants

Twenty healthy university students participated in the study (11 males, 9 females, age: 22.0±1.7 years, age range: 19–25 years). All the participants were right-handed, non-smokers and free of any mental or somatic disorder or substance/medication use. All the participants maintained a regular sleep–wake schedule before and during the study, as documented by sleep diaries. The participants were recruited from the community and compensated for participation. The study was approved by the local Ethics Committee at the University Medical Center Freiburg and was conducted in accordance with the Declaration of Helsinki. All the participants provided written informed consent before participation.

### Study design

**Figure 6:**
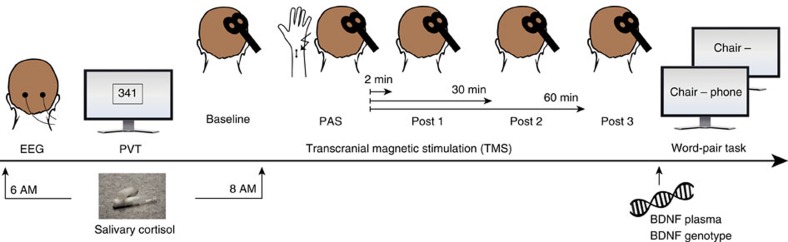
Electrophysiological, behavioural and molecular assessments. Measurements started at 6:45 AM with the wake electroencephalography (EEG) and Psychomotor Vigilance Task (PVT). At 0800, h, the TMS protocol was started consisting of a baseline measurement followed by paired associative stimulation (PAS) and three post measurements (2, 30 and 60 min after the end of PAS). Salivary cortisol was assessed at 0600, h and 0800, h. After the TMS measurement, 15 ml of blood was taken to determine brain-derived neurotrophic factor (BDNF) plasma concentration and BDNF genotype. Last, a declarative memory task (word-pair task) was conducted.

The participants underwent a screening session and a repeated-measures electrophysiological, behavioural and molecular study protocol (Fig. 6) after one night of normal nighttime sleep at home (self-reported total sleep time 6.7±0.6 h, range 5.4–7.5 h) and one night of total sleep deprivation (T1 and T2). At screening, the participants obtained a sleep diary for 2 weeks to monitor sleep–wake behaviour across the study. T1 and T2 took place seven and 14 days, respectively, after screening. The night before T1 and T2, participants either slept about 7 h at home or underwent one night of total sleep deprivation at the Department of Psychiatry and Psychotherapy with standardized activities and continuous supervision by staff members. The order of the sleep and sleep deprivation condition was counterbalanced. Screening comprised the Composite International Diagnostic Interview to exclude any mental disorders and the Beck Depression Inventory. The Edinburgh Handedness Inventory was used to ensure that all the participants were right-handed. A TMS screening comprised questions concerning the safety of TMS. A few magnetic stimuli were administered to the primary motor cortex (M1) to ensure that TMS was applicable. The Pittsburgh Sleep Quality Index and the Epworth Sleepiness Scale were used to detect any sleep-related pathology.

### Indices of net synaptic strength

**Figure 7:**
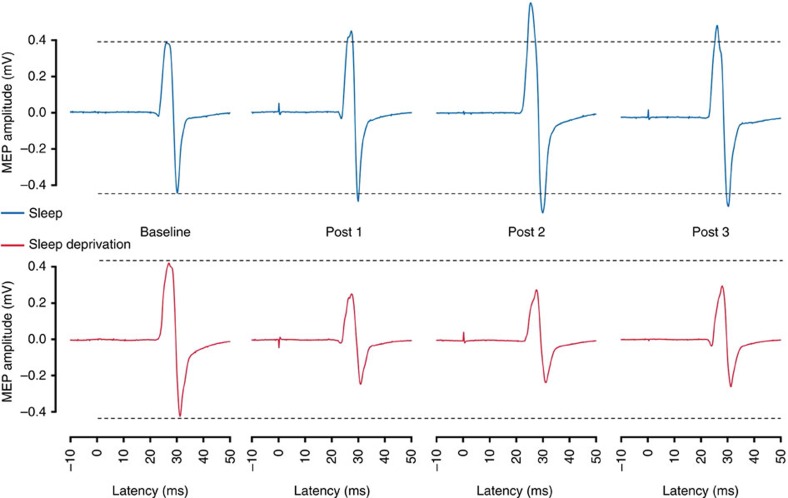
Raw examples of single motor-evoked potentials (MEPs). Single MEPs of one participant were selected to illustrate the results. MEP amplitudes increased from baseline level after paired associative stimulation (PAS) in the sleep condition, whereas MEP amplitudes decreased from baseline level after PAS in the sleep deprivation condition.

TMS was applied using a figure-of-eight stimulation coil with an outer diameter of 90 mm that was centred tangentially on the skull over the right primary motor cortex (M1) with the handle pointing in a posterior direction and laterally at an angle of 45° away from the midline. The coil was connected to a Magstim 200 stimulator (The Magstim Company Ltd., Whitland, UK) with a monophasic current waveform. By moving the coil over M1 while administering stimuli of suprathreshold intensity at 0.25 Hz, the optimal coil position for eliciting MEPs of maximal amplitude of the left APB muscle was identified (‘hotspot'). The coil position was recorded using a stereotaxic, optically tracked navigation system to reduce experimenter bias, consisting of a camera (Polaris Vicra P6, NDI, Waterloo, Ontario, Canada), custom-made software (BrainView, Fraunhofer Institute, Stuttgart, Germany) and passive sphere markers^47^, and kept constant throughout the measurements. RMT was determined using a maximum-likelihood threshold-hunting paradigm^48^ that consisted of 16 TMS stimuli at 0.25 Hz starting at 45% MSO. The stimulation intensity was adjusted at the beginning of each condition (sleep, sleep deprivation) to elicit MEPs with peak-to-peak amplitudes of on average 600–1,400 μV (SI_1mV_) and was kept constant throughout all measurements within each condition to assess changes in mean MEP amplitudes. To adjust stimulation intensity, 20 MEPs were recorded with a stimulation intensity of 120% RMT. If the mean amplitude of the 20 MEPs was <600 μV or >1,400 μV, the stimulation intensity was increased or decreased, respectively, and another 20 MEPs were recorded until the correct intensity was determined. At each TMS measurement (baseline, post 1, post 2, post 3), 20 TMS pulses were administered at a frequency of 0.1 Hz. The MEP amplitudes were determined by measuring the two highest peaks of opposite polarity. Raw examples of individual MEP traces are presented in Fig. 7. For each TMS measurement (baseline, post 1, post 2, post 3), the mean MEP amplitude was calculated by averaging the individual peak-to-peak amplitudes of the 20 TMS pulses. To estimate cortical excitability, we compared the stimulation intensity adjusted to elicit a mean MEP amplitude of about 1 mV peak-to-peak (SI_1mV_) in each condition before PAS between the two conditions.

MEPs were recorded from the left APB muscle using silver/silver chloride electrodes (AMBU, Ballerup, Denmark) in a belly-tendon montage. The participants were instructed to relax the target muscle during all measurements. The overall EMG level before the application of a TMS stimulus was near zero and did not differ between the conditions. The EMG signals were band-pass filtered (20–2,000 Hz) and amplified using an Ekida DC universal amplifier (Ekida GmbH, Helmstadt, Germany), digitized at a 5 kHz sampling rate using a MICRO1401*mk*II data acquisition unit (Cambridge Electronic Design Ltd., Cambridge, UK) and stored on a computer for online visual display and later offline analysis using Signal Software version 3 (CED Ltd, UK). MEPs were excluded from the analysis if the muscle activity exceeded a value of 0.05 mV in a time frame 100 ms before the TMS stimulus. Very few MEPs were excluded (sleep condition: 0.75%, 12 out of 1,600; sleep deprivation condition: 0.44%, 7 out of 1,600).

The wake EEG was recorded from the electrode positions O1 and O2 referenced to Fpz according to the 10–20 system^49^ using a Neuroscan SynAmps amplifier (Compumedics Neuroscan, Charlotte, NC, USA). The EEG recordings were performed for a 2.5 min period of sustained wakefulness and the average spectral power in the EEG theta frequency band (3.5–8 Hz) of artifact-free 2 s epochs was calculated.

### Indices of LTP-like plasticity

The PAS protocol closely followed standard procedures^19^. PAS consisted of 200 pairs of peripheral and cortical stimuli at a frequency of 0.25 Hz. Peripheral electrical stimulation of the median nerve at the left wrist was followed by TMS of the right M1 at the optimal site to elicit MEPs in the left APB muscle with an ISI of 25 ms. Electrical stimulation was applied through a Digitimer DS7 electrical stimulator (Digitimer Ltd., Welwyn Garden City, Hertfordshire, UK) at the optimal stimulation site at the wrist using a bipolar electrode with the cathode proximal. Stimuli were constant current square wave pulses with a duration of 1,000 μs at an intensity of 300% of the sensory perceptual threshold. TMS intensity was set at SI_1mV_ as determined before PAS. Taking into account that the level of attention may influence PAS effectiveness^50^, the participants were instructed to direct their attention to the stimulated hand and count silently the number of randomly administered electrical stimuli applied to the thumb of the stimulated hand using a second bipolar electrode (200% perceptual threshold, constant current square wave pulses, duration 200 μs, cathode proximal). Four electrical stimuli were applied at the midpoint of the interval between successive paired stimuli during the stimulation protocol. The participants were asked to report the number of stimuli after the PAS intervention. As an index of LTP-like plasticity, we analysed the change in MEP amplitude after the PAS intervention (post 1–3) compared with pre-PAS.

In the word-pair task, 46 semantically unrelated word-pairs were randomly presented on a computer screen using the Presentation software. Each word-pair was presented for 5,000 ms, separated by an ISI of 1,000 ms. Presentation of the word-pairs was immediately followed by cued recall, that is, participants were required to name the second word upon presentation of the first word. Correct and incorrect responses were noted by staff members without feedback. Three presentation/recall trials were conducted. Recall performance was assessed as the number of correctly recalled words in each trial. To control for primacy and recency effects, four additional word-pairs at the beginning and at the end were not included in the analysis. Parallel versions of the task were used for repeated measurements.

### Potential modulators of synaptic plasticity

BDNF has emerged as an important modulator of synaptic plasticity^51^. We analysed the BDNF genotype and plasma levels. Genomic DNA was purified from 3 ml of whole blood. Fifty nanograms of genomic DNA was used to amplify a 281 bp polymerase chain reaction (PCR) product surrounding the site of the Val66Met polymorphism for subsequent direct sequencing by GATC Biotech in Konstanz, Germany. Direct sequencing was performed with 3.2 pmol of the reverse primer used for initial PCR amplification. The genotype of each participant was determined following two independent rounds of direct sequencing^52^. The following primers were used: 5′-CAGGTGAGAAGAGTGATGACCA-3′ (forward) and 5′-GCATCACCCTGGACGTGTAC-3′ (reversed). BDNF plasma levels were measured by *E*_max_ ImmunoAssay Systems ELISAs (G7610) according to the manufacturer's instruction.

To control for possible effects of cortisol levels, saliva specimens were collected at 0600, h (before breakfast) and 0800, h (1 h after breakfast) at T1 and T2. All the samples were stored at −20 °C until the analysis at the BioInnovationsZentrum Dresden, Germany. After thawing, salivettes were centrifuged at 3,000*g* for 5 min, which resulted in a clear supernatant of low viscosity. Salivary concentrations were measured using a commercially available chemiluminescence immunoassay with high sensitivity (IBL International, Hamburg, Germany). The intra- and interassay coefficients of variation for cortisol were below 8%.

To control for possible effects of vigilance, the PVT was used. The participants were instructed to press a response button as fast as possible to stop a visual millisecond (ms) counter on a computer screen starting at a variable ISI of 2,000–10,000 ms. In response to the reaction, the counter display stopped, allowing the participant to read the reaction time (RT) for 1,000 ms before restart. The total test duration was 10 min. Mean response speed (1,000 ms per RT; 100 ms ≤RT<500 ms) and number of lapses (errors of omission; RTs ≥500 ms) were analysed.

### Data analysis

IBM SPSS 21 was used for statistical analysis. The data are reported as means±s.d.s, if not indicated otherwise. To test our first hypothesis of decreased inducibility of LTP-like plasticity after sleep deprivation compared with sleep, a 2 × 4 rm-ANOVA with the within-subject factors Condition (sleep, sleep deprivation) and Time (baseline, post 1, post 2, post 3) and MEP amplitudes as the primary end point was conducted. Power calculation was done for this analysis (*F* test with repeated measures, G*Power 3.1.9.2). To compare memory performance as a secondary index, a 2 × 3 rm-ANOVA with the within-subject factors Condition (sleep, sleep deprivation) and Session (sessions 1–3) and the number of correctly recalled word-pairs in each trial as the dependent variable was conducted. *Post hoc* paired-sample *t*-tests were conducted for significant rm-ANOVA effects. To test our second hypothesis of increased cortical excitability/net synaptic strength after sleep deprivation compared with sleep, a paired-sample *t*-test was used to compare TMS intensity to elicit MEPs of about 1 mV peak-to-peak (SI_1mV_) as the primary end point. To compare EEG theta power (3.5–8 Hz) as a secondary index, a paired-sample *t*-test was used. Partial eta square values (*η*_p_^2^) were calculated as effect sizes for ANOVAs (low: <0.06; medium: ≥0.06 and <0.14; large: ≥0.14). The level of statistical significance was set at *P*<0.05 (two-tailed).

### Data availability

The data that support the findings of this study are available from the corresponding author upon request.

## Additional information

**How to cite this article:** Kuhn, M. *et al*. Sleep recalibrates homeostatic and associative synaptic plasticity in the human cortex. *Nat. Commun.* 7:12455 doi: 10.1038/ncomms12455 (2016).

## Supplementary Material

Supplementary Information Supplementary Table 1
