# Supplementary material for: Sleep recalibrates homeostatic and associative synaptic plasticity in the human cortex
Source: Nat Commun. 2016 Aug 23;7:12455. doi: 10.1038/ncomms12455 (PMC4996971; doi:10.1038/ncomms12455)
Supplement: Supplementary Information — Supplementary Table 1 [file ncomms12455-s1.pdf]

Supplementary Table 1. Mean amplitude of motor evoked potentials (mV) before (baseline) and after (post) paired associative stimulation (PAS).

	**Sleep**	**Sleep Deprivation**	***t***	***p***
Baseline	0.82 ± 0.21	0.85 ± 0.16	-0.5	.624
Post 1 (2 min after PAS)	0.90 ± 0.36	0.72 ± 0.28	2.0	.062
Post 2 (30 min after PAS)	0.99 ± 0.33	0.73 ± 0.20	3.8	**.001**
Post 3 (60 min after PAS)	0.92 ± 0.35	0.76 ± 0.35	1.7	.103

Data represent means ± standard deviations (n = 20). Paired samples t-tests were used (two-tailed).

Significant results are given in bold.
